# A Bystander Effect of Lung Cancer Chemotherapy on Chronic Echinococcal Disease

**DOI:** 10.14740/wjon920w

**Published:** 2015-08-27

**Authors:** Saroja D. Bangaru, Phyllis E. Kozarsky, Daniel J. Lee, Gabriel L. Sica, Taofeek K. Owonikoko

**Affiliations:** a Department of Medicine, Emory University School of Medicine, Atlanta, GA, USA; b Division of Infectious Diseases, Emory University School of Medicine, Atlanta, GA, USA; c Department of Radiology, Emory University School of Medicine, Atlanta, GA, USA; d Department of Pathology, Emory University School of Medicine, Atlanta, GA, USA; e Department of Hematology & Medical Oncology, Emory University School of Medicine, Atlanta, GA, USA

**Keywords:** Lung cancer, Echinococcal, Chemotherapy, Pemetrexed, Paclitaxel, Albendazole

## Abstract

Hydatid cystic disease is a parasitic infestation caused by *Echinococcus granulosus* and commonly manifests as hepatic and pulmonary cysts. When feasible, based on cyst size and location, surgical resection is potentially curative. Post-surgical recurrence of disease is encountered in up to 25% of patients. Secondary peritoneal contamination is a recognized complication in 5-10% of cases. Disseminated disease is usually palliated using systemic anti-parasitic agents such as benzimidazoles, albendazole and mebendazole but worsening of disease post-systemic treatment is frequent in 14-25% of patients. In this report, we share our experience of a patient with long-standing, chronic disseminated hydatidosis and subsequent diagnosis of non-small cell lung cancer who manifested evidence of reduced activity of the echinococcal disease following institution of chemotherapy for his new diagnosis of lung cancer. There was significant reduction in the serum level of anti-echinococcal antibody titers in tandem with chemotherapy administration. There was also minimal but notable decrease in the size of the cysts on serial cross-sectional imaging obtained for monitoring cancer response to chemotherapy. This intriguing observation of a possible benefit of anticancer chemotherapy against echinococcal disease in this index case may provide new insights for therapeutic exploration in disseminated echinococcal disease.

## Introduction

Cystic echinococcosis is a zoonotic disease caused by the parasitic cestode *Echinococcus granulosus*. While rare in the US and Western Europe, echinococcal disease is endemic in the Middle East and Mediterranean regions and South America where the incidence is as high as 220 per 100,000 [1]. Human infection occurs via accidental ingestion of the ova in the feces of infected dogs and sheep. Larval growth and penetration of the intestinal mucosa permits entry into the bloodstream via the portal circulation. As a result, the liver is the most frequent site of infection (50-70%), followed by the lung (10-40%), and then the brain and other viscera [2, 3]. Thus the disease is frequently clinically silent until cysts are large enough to cause mass effect, resulting in abdominal pain or palpable masses. Complicated cystic disease is associated with significant morbidity related to cyst rupture such as fever, pruritus, and potentially fatal anaphylaxis. Local effect may also cause common bile duct obstruction with jaundice while thoracic involvement can present with unexplained chest pain or hemoptysis [4].

Hydatidosis is diagnosed based on clinical characteristics along with imaging and confirmatory immunodiagnostic studies (ELISA, IFAT, immunoblot, etc.). Abdominal ultrasound is the gold standard for diagnosing peri-hepatic cysts because of low cost and availability in addition to ability to characterize extent of disease. The serologic antibody titer, IgG-ELISA, has high sensitivity of approximately 95% in diagnosing echinococcal disease but it is comparatively non-specific and therefore, has limited use in confirmation of diagnosis or tracking disease activity in response to treatment [5, 6]. Due to the risk of peritoneal contamination and of anaphylactic reaction, cyst fluid aspirate collection for PCR-based diagnostic antigen test and microscopic examination is less frequently employed and only in cases of diagnostic uncertainty [7]. Peritoneal contamination due to microrupture of cysts or leakage during abdominal surgery complicates up to 10% of cases of echinococcal disease. Disseminated disease is more challenging to treat and difficult to eradicate, with a very high risk of recurrence [8].

There is currently no consensus on the best treatment approach for echinococcal hydatidosis [9]. Management options include surgical resection, percutaneous drainage, and systemic anti-parasitic therapy. The choice of treatment is determined by the patient’s fitness for surgery, the number, size, and location of the cysts as well as the risk of associated complications [10]. Surgical resection is a definitive and curative treatment modality in appropriate patients [3, 11]. However, there is a very high rate of recurrence post-surgery ranging 2-25% [9, 12]. Surgery is not recommended in patients with multiple small cysts, calcified cysts, or cysts located in poorly accessible areas of the body. Percutaneous drainage and instillation of a scolicidal agent intraoperatively, commonly referred to as PAIR, is an alternative, minimally invasive but more technically challenging method that is comparable to standard cystectomy in efficacy [13, 14].

Systemic anti-parasitic therapy is used for short-term peri-interventional treatment and in cases of inoperable liver echinococcosis or multiple organ involvement [15]. The benzimidazole derivatives, albendazole and mebendazole, are the only agents approved in the treatment of echinococcosis. These agents bind to beta-tubulin, thereby preventing microtubule polymerization, which are essential for energy metabolism leading to impaired energy metabolism and death of adult echinococcal parasites and eggs. According to the 2001 WHO Summary on Echinococcal Disease, over 2,000 documented cases had been treated with benzimidazoles mostly for patients with inoperable cystic echinococcal disease. Benzimidazole therapy for up to 12 months achieved complete disappearance of cysts in 10-30% of patients, cyst degeneration or reduction in cyst size (indicating improvement) in 50-70% of patients, and no morphologic change in cysts (indicating lack of clinical response) in the remaining 20-30% of patients [16]. The modest curative success of systemic therapy in only 30% of patients underscores the need for new options of treatment in this disease. We therefore share our interesting observation of a potential benefit of cytotoxic chemotherapy against echinococcal disease in a 68-year-old male with chronic peritoneal cystic echinococcosis and subsequent diagnosis of stage IIIB, non-small cell lung cancer initially treated with curative intent using external beam radiation therapy and chemotherapy. He was subsequently treated with different palliative chemotherapy agents following progression of his lung cancer.

## Case Report

**Figure 1:**
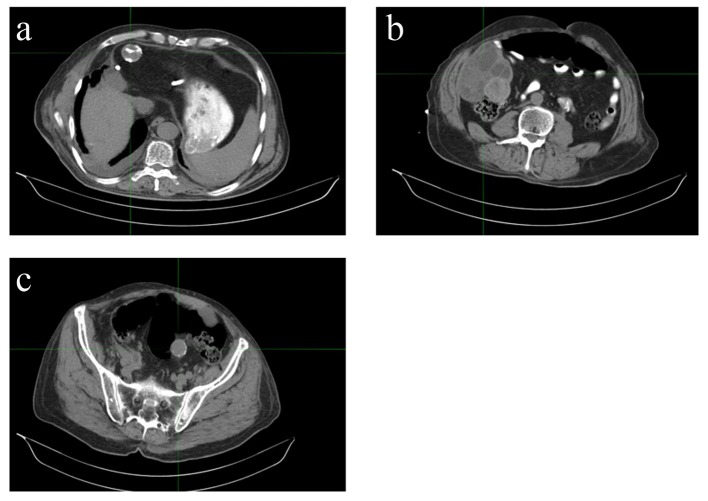
Disseminated cystic echinococcosis in our index patient, demonstrating hepatic (a), peritoneal (b), and pelvic (c) disease.

**Figure 2:**
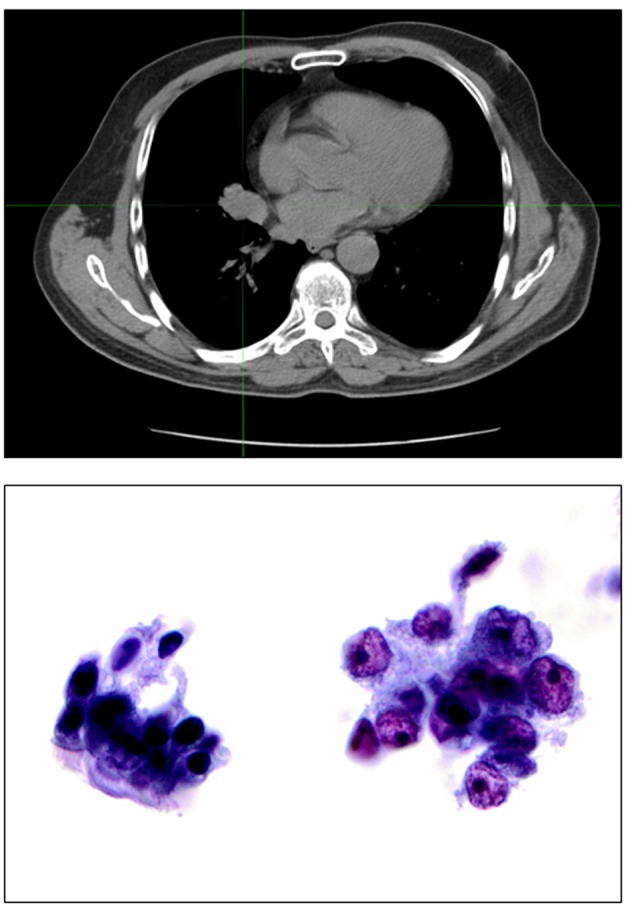
Top: right hilar mass corresponding to the unresectable stage T4 lung cancer. Bottom: bronchial brushing stained by Papanicolaou staining, × 400. Cluster of benign bronchial cells with cilia for comparison (left). Malignant cells with irregular nuclear membranes and prominent nucleoli compatible with non-small cell lung cancer, favor adenocarcinoma (right).

**Figure 3:**
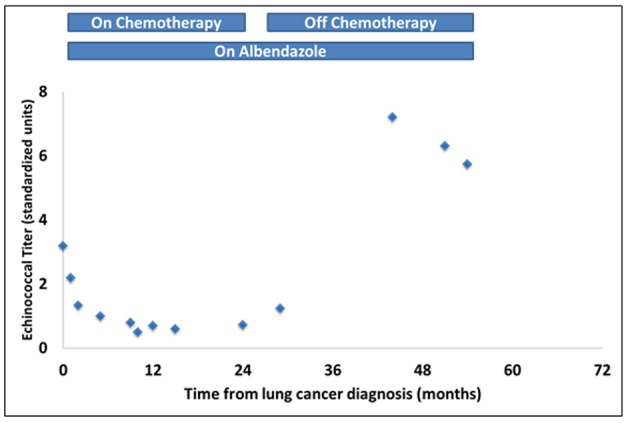
Changes over time in serum levels of echinococcal antibody titers measured by IgG ELISA. Note the downward trend in titer levels following initiation of chemotherapy and radiation and a rise above the baseline levels after completion of chemoradiation.

The patient is a 68-year-old male, immigrant to the US from Iraq, where he was a sheepherder. He has a long-standing medical history of echinococcal hydatidosis and known hepatic cystic disease for more than 30 years. He had been treated previously with cystectomies and hepatic lobectomy in his native country. Prior surgical treatment was complicated by intraoperative contamination and dissemination of the disease to the peritoneum and pelvis secondary (Fig. 1). Repeat exploratory laparotomy for palliative control of the extensive peritoneal and pelvic cystic disease was not curative and he was placed on lifetime maintenance therapy with albendazole. Serum titers against echinococcal antigen by an IgG-ELISA and cross-sectional imaging when necessary were employed to monitor disease status and response to therapy. Patient also has a 50-pack-year smoking history but otherwise has an unremarkable medical history. A routine abdominal CT scan obtained in late 2008 to monitor disease status revealed an incidental finding of a right lung base opacity. Dedicated chest CT confirmed a 2.5 × 1.6 cm right lower lobe nodule along the infrahilar region with possible post-obstructive atelectasis (Fig. 2). Whole body PET scan showed high FDG uptake in the perihilar soft tissue mass with SUV of 7, consistent with primary bronchogenic carcinoma. There were no foci of distant tumor metastasis but FDG-avid hydatid cysts were noted in the peritoneal and pelvic cavities. A biopsy of the lung lesion obtained via fiber optic bronchoscopy confirmed the presence of non-small cell lung cancer, favoring adenocarcinoma (Fig. 2) with clinical staging of T4N0M0 (AJCC 6th edition). Thoracoscopic resection was attempted but aborted due to intraoperative finding of direct pericardial invasion by the tumor. The patient was treated with chemotherapy and radiation using a sequential approach due to significant postoperative debility that made concurrent therapy unsafe. Serial measurement of echinococcal serum antibodies with an IgG ELISA assay (ARUP Laboratories, Salt Lake City, UT) was performed to monitor disease activity. The patient received three cycles of systemic chemotherapy with cisplatin and docetaxel over a 3-month period followed by radiation therapy to the chest to a total dose of 66.6 Gy in 37 fractions and tolerated treatment with minimal toxicity. The most proximal echinococcal titer obtained prior to initiation of chemotherapy was 3.2. The repeat titer after completing two cycles of chemotherapy showed a drop in the titers to 2.2 down reaching a nadir of 1.8 (Fig. 3). A repeat measurement of echinococcal titer about 2 months into radiation treatment showed a nadir level of 1.0. Restaging scan including an abdominal CT scan after completion of chemoradiation was stable with a minimal decrease in the size of index intra-abdominal echinococcal cysts. Post-radiation, the patient received consolidation chemotherapy with three cycles of carboplatin and docetaxel and achieved complete response in the lung mass along with a further drop in the echinococcal titer to 0.5, the lowest value ever measured over the 10 years that the patient had been monitored for his chronic echinococcal infection at our center. Monitoring for disease recurrence with restaging scans continued while the patient was off chemotherapy. Although he continued to receive albendazole for his echinococcal disease during this time, there was a gradual increase in size of some of the areas of perihepatic echinococcal cystic lesion.

Following recurrence of his cancer approximately 10 months later, patient was restarted on chemotherapy using a combination of carboplatin and pemetrexed. There was a similar reduction in the echinococcal titer with the resumption of chemotherapy. Due to treatment-related side effect, the patient elected to discontinue further chemotherapy following which the antibody titer gradually increased, reaching a peak of 6.32 within 4 months of the last dose of chemotherapy despite continued maintenance on albendazole. The patient is now approximately 6 years out from his original diagnosis of locally advanced lung cancer and remains alive and of chemotherapy.

## Discussion

Cystic hydatidosis is a rare parasitic disease associated with significant morbidity and diminished quality of life. Treatment of localized disease is infrequently curative while symptom palliation and control of disease remain the achievable goals of systemic treatment for disseminated infection. Our experience with this index patient with peritoneal hydatidosis and concomitant lung cancer is intriguing because of the observed reduction in markers of echinococcal activity and the administration of chemotherapy. The echinococcal IgG titer is regarded as the gold standard for assessing disease burden and activity [5]. The significant drop in echinococcal titers during active chemotherapy treatment and noticeable decrease in the size of the cysts on abdominal CT therefore raised the possibility of some anti-parasitic effect of the chemotherapy agents administered for the treatment of patient’s lung cancer. Given the limited options for effective treatment of echinococcal disease, this intriguing clinical observation provides some insight on potential avenues to explore for novel treatment of echinococcal disease. Although this is the first report of the potential activity of cytotoxic chemotherapy against echinococcal parasitic disease in the clinical setting, there have been previous preclinical studies showing *in vitro* and *in vivo* efficacy of chemotherapeutic agents against echinococcus [17-19].

The cross-species activity of cytotoxic anticancer agents is plausible and perhaps not surprising. Neoplastic cells and echinococcal cellular subunits are similar in their increased proliferative capacity and in their metastatic ability that is dependent on secreted proteolytic enzymes [20]. There is also mechanistic similarity with regard to intracellular microtubule as the target of taxanes employed for lung cancer therapy and the benzimidazole, which is an established treatment of echinococcal disease target. Both classes of agents cause microtubule dysfunction. The noticeable reduction in echinococcal disease titers with cytotoxic chemotherapy when there was no further drop in the titer with preceding albendazole therapy suggests that cytotoxic chemotherapy agents may be worth exploring especially in patients with recalcitrant cystic disease that is no longer responsive to standard anti-parasitic therapies. Given the vastly different risk-benefit balance of chemotherapy administered to treat cancer as opposed to treatment of echinococcal disease, significant modification of the dose and schedule of administration to minimize toxicity and improve convenience will be important. It is conceivable that a dose of chemotherapy much lower than is necessary for cancer therapy would be sufficient for meaningful control of echinococcal disease. This is akin to the established use of much lower doses of cytotoxic agents like methotrexate, cyclophosphamide and azathioprine as immunomodulatory agents in the management of rheumatoid arthritis and lupus erythematosus [21, 22].

Another interesting angle in this case is the prolonged survival past 6 years post-diagnosis recorded in this patient with advanced stage lung cancer despite the fact that he only received two different lines of chemotherapy regimens, i.e. platinum doublet and single agent pemetrexed. The median survival for patients with stage IIIB non-small cell lung cancer treated with chemotherapy and radiation is approximately 20 months [23, 24]. Whether the chronic activation of the immune system by his echinococcal disease contributed to this outcome is only a mere conjecture.

However, it has been previously noted that active infection can modulate the course of cancer. An idea was first mooted in the mid-1800s based on the observation of a high rate of regressions of soft tissue sarcoma in patients whose disease was complicated by acute streptococcal infections [25, 26]. Similar observations were later reported in the mid-1900s in a series of retrospective studies demonstrating an association between febrile illness and spontaneous regression of cancer [25]. Further support came from mouse models showing spontaneous regression of transplanted tumors in mice infected with various infectious agents such as toxoplasma, *Listeria monocytogenes*, and *Corynebacterium parvum* [27, 28]. There is also evidence of a cytotoxic immune response in the setting of acute infection leading to suppression of tumor growth in part through the action of interferon gamma, a cytokine produced by Th1 helper cell [29-31]. There also could be some synergistic effect of chemotherapy and albendazole given the overlapping mechanisms of action. Indeed, preclinical work in lung cancer models showed a synergistic anticancer activity between niclosamide and erlotinib, a kinase inhibitor of epidermal growth factor receptor [32, 33].

In conclusion, we report an interesting observation of potential efficacy of established anticancer chemotherapy agents against echinococcal disease. Serendipitous observations such as this have the potential to stimulate novel approaches to the treatment of patients with recalcitrant echinococcal disease.
